# Supplementary figures and images for: Selection and validation of reference genes for RT-qPCR in ophiocordyceps sinensis under different experimental conditions
Source: PLoS One. 2024 Feb 6;19(2):e0287882. doi: 10.1371/journal.pone.0287882 (PMC10846742; doi:10.1371/journal.pone.0287882)

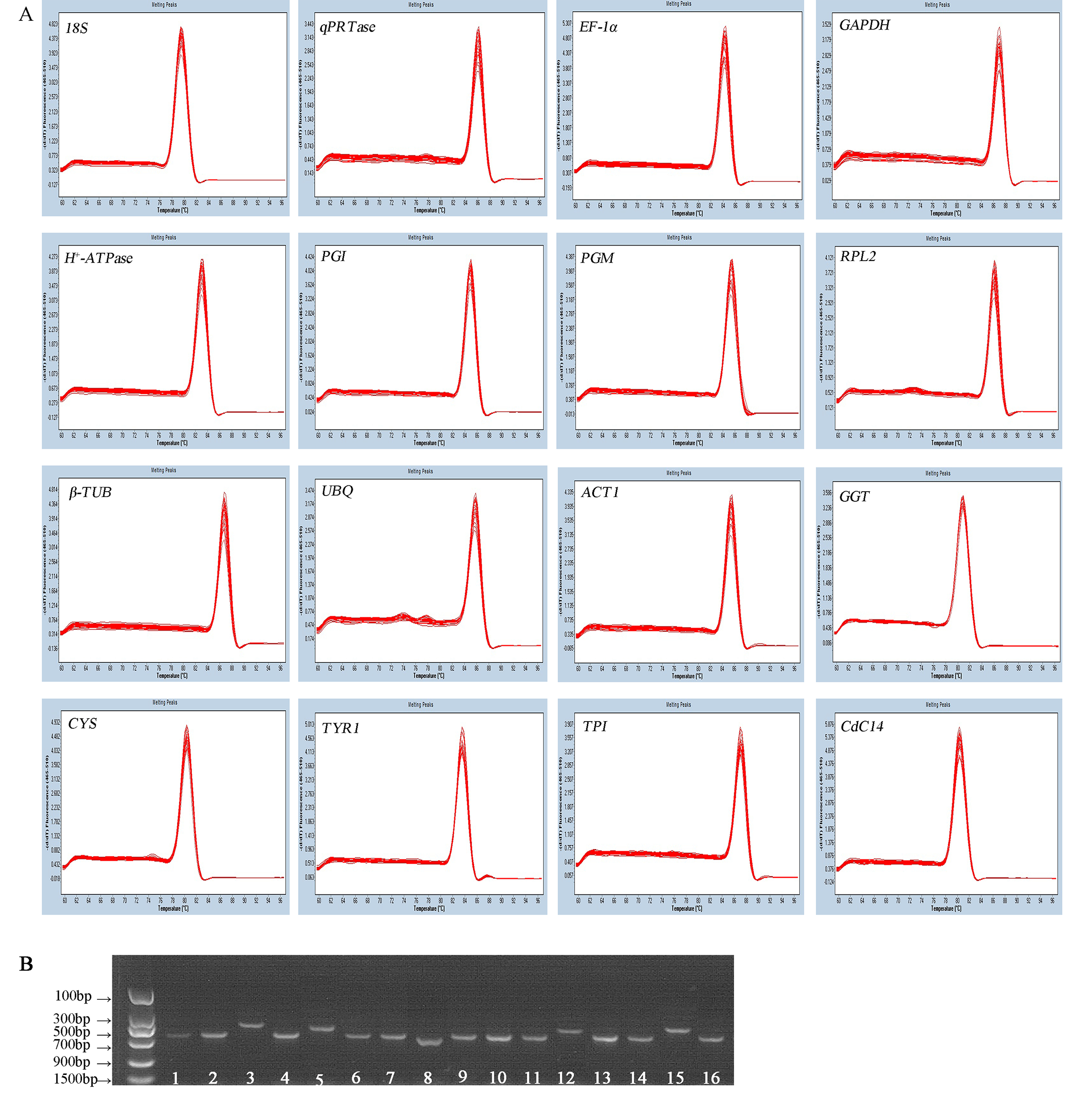

Supplement: S1 Fig — A. Dissolution curves and amplification products and of sixteen CRGs. B. Amplifcation products of 16 CRGs: DNA marker, DL2000; 1–16, 18S rRNA, QPRTase, β-TUB, RPL2, EF1-α, PGI, PGM, H+-ATPase, ACT1, UBQ, GAPDH, CYS, GGT, TPI, TYR1and CDC14. (TIF) [file pone.0287882.s001.tif]
