# Supplementary material for: Selection and validation of reference genes for RT-qPCR in ophiocordyceps sinensis under different experimental conditions
Source: PLoS One. 2024 Feb 6;19(2):e0287882. doi: 10.1371/journal.pone.0287882 (PMC10846742; doi:10.1371/journal.pone.0287882)
Supplement: S1 Table — (PDF) [file pone.0287882.s002.pdf]

S 1 Table. the optimal combination of reference genes under different conditions

	DD	DM	DS	Total
V2/3	0.051	0.0799	0.114	0.132
V3/4	0.0456	0.0821	0.119	0.126
V4/5	0.0498	0.0845	0.122	0.119
V5/6	0.0466	0.0856	0.109	0.102
V6/7	0.0433	0.0817	0.099	0.098
V7/8	0.0432	0.0800	0.094	0.091
V8/9	0.0479	0.0689	0.082	0.081
V9/10	0.0417	0.0671	0.086	0.085
V10/11	0.0411	0.0645	0.071	0.077
V11/12	0.0359	0.0633	0.066	0.081
V12/13	0.0405	0.0645	0.069	0.085
V13/14	0.0419	0.0655	0.072	0.080
V14/15	0.0422	0.0723	0.073	0.079
V15/16	0.0443	0.0739	0.070	0.083
