# Supplementary material for: Selection and validation of reference genes for RT-qPCR in ophiocordyceps sinensis under different experimental conditions
Source: PLoS One. 2024 Feb 6;19(2):e0287882. doi: 10.1371/journal.pone.0287882 (PMC10846742; doi:10.1371/journal.pone.0287882)
Supplement: Attachment — Submitted filename: renamed_04a61.docx [file pone.0287882.s004.docx]

**Reponse to the journal requirements and reviewer(s)**

**Reponse to journal requirements**

**Question 1:** Please review your reference list to ensure that it is complete and correct. If you have cited papers that have been retracted, please include the rationale for doing so in the manuscript text, or remove these references and replace them with relevant current references. Any changes to the reference list should be mentioned in the rebuttal letter that accompanies your revised manuscript. If you need to cite a retracted article, indicate the article’s retracted status in the References list and also include a citation and full reference for the retraction notice.

**Answer 1:** Thank you for your good advice,I have check the reference list of my manuscript. All cited papers were complete and correct except for reference 20, due to this paper was a Chinese journal, so it cannot be identified,while this cited paper was important for the background construction.

**Reponse to Additional Editor Comments:**

**Question 1:** This manuscript was revised well. However, I found some references were cited wrong. For example, RefFinder program was developed by Dr. Zhang's lab, please see the publications about this RefFinder program: Xie, F., Wang, J. & Zhang, B. RefFinder: a web-based tool for comprehensively analyzing and identifying reference genes. Funct Integr Genomics 23, 125 (2023). <https://doi.org/10.1007/s10142-023-01055-7>; and Xie F, Xiao P, Chen D, Xu L, Zhang B (2012) miRDeepFinder: a miRNA analysis tool for deep sequencing of plant small RNAs. Plant Mol Biol 80(1):75–84.<https://doi.org/10.1007/s11103-012-9885-2.>Please correct this references and give the credit to the right person. Please also carefully check other references.

**Answer 1:** I am sorry for my ignore. Firstly, I have replaced references 25 with your advised (<https://doi.org/10.1007/s10142-023-01055-7>).Then, I have also carefully check the other references, I found reference 10 was unsuitable and it has been replaced.

**Question 2:**While revising your submission, please upload your figure files to the Preflight Analysis and Conversion Engine (PACE) digital diagnostic tool, <https://pacev2.apexcovantage.com/>. PACE helps ensure that figures meet PLOS requirements

**Answer 2:** The image format detection of Fig 1, Fig 2 and S1 Fig have been carried out with he Preflight Analysis and Conversion Engine (PACE) digital diagnostic tool, among which,Fig 1 reached figure file requirements, Fig 2 and S1Fig have been revised and upload again.
